# The Supratrochlear Artery Revisited: An Anatomic Review in Favor of Modern Cosmetic Applications in the Area

**DOI:** 10.7759/cureus.7141

**Published:** 2020-02-29

**Authors:** Loukas Agorgianitis, Eleni Panagouli, George Tsakotos, Gregory Tsoucalas, Dimitrios Filippou

**Affiliations:** 1 Anatomy and Surgical Anatomy, National and Kapodistrian University of Athens, Athens, GRC; 2 Anatomy, National and Kapodistrian University of Athens, Athens, GRC; 3 Anatomy, School of Medicine, National and Kapodistrian University of Athens, Athens, GRC; 4 Anatomy, School of Medicine-Democritus University of Thrace, Alexandroupolis, GRC; 5 Surgery, School of Medicine, National and Kapodistrian University of Athens, Athens, GRC

**Keywords:** tissue filler injections, ophthalmic artery

## Abstract

The supratrochlear artery represents a terminal branch of the ophthalmic artery. Cosmetic interventions may traumatize it, resulting in a circulation in the lesion in glabellar region and in the medial aspect of the forehead.

This review article aims to synopsise the existing knowledge of the anatomy of the supratrochlear artery in close correlation with minimally invasive cosmetic procedures in the facial area such as soft-tissue filler injections. Their possible adverse effects and their safe application based on the topographic anatomy were included.

A literature review was performed in PubMed/Medline online medical database.

The superficial course of the supratrochlear artery, as well as the rich, variable anastomotic network that it forms with the supraorbital, angular and dorsal nasal artery raise clinical questions in the case of soft-tissue filler injections in the nasoglabellar and central forehead area. Accidental cannulation of the supratrochlear artery and ultimately, the risk of embolization of the central retinal artery in a retrograde fashion might lead to injury with questionable cosmetic results.

Although the risk of complications from the use of soft tissue fillers is considered rare, once happen, the results could be devastating for the quality of life. Thus, the comprehension of the anatomy of the supratrochlear artery is paramount for the health practitioners.

## Introduction and background

The supratrochlear artery (STA) is a terminal branch of the ophthalmic artery (OA) which in turn is the first intracranial branch of the internal carotid artery (ICA) [1]. After exiting the orbit, the vessel travels vertically, ascending to the forehead, approximately 2 cm from the midline [2]. Despite its short course, accurate knowledge of the anatomy of the STA is of crucial importance. Statistical analyses from the American Society of Plastic Surgeons, demonstrate that there is an increasing demand for minimally invasive cosmetic procedures in facial area [3]. Alongside, a certain number of adverse effects was observed varying from mild local skin irritation to vision loss and even cerebral infarction soon after the application of cosmetic interventions in the area [4,5]. The purpose of this study is to present a review of the available literature concerning the STA's anatomy in correlation with its clinical significance in cosmetic medical procedures.

## Review

Study design

**Figure 1:**
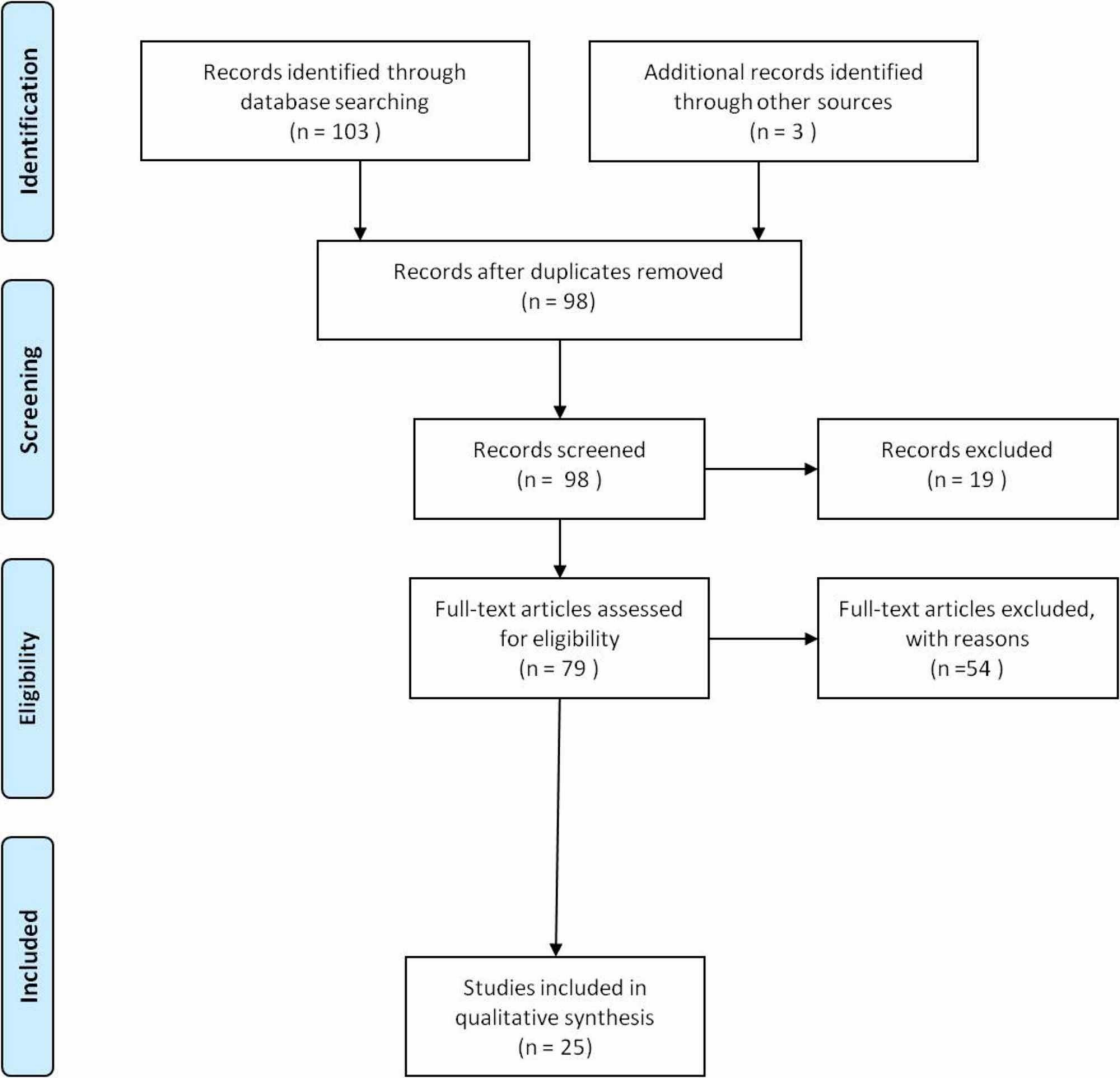
Prisma flow diagram of the studies The diagram describes the steps followed during the selection of the eligible studies.

A bibliographic research in PubMed medical data base for studies on supratrochlear artery and its clinical relevance was performed. The following search terms were used: “supratrochlear artery vascular anatomy”, “supratrochlear artery vascular anatomy variations”, “supratrochlear artery and filler injections” and “filler injections and vision loss”. Inclusion criteria were, i) English language, ii) suitable methodology of adverse effects diagnosis, while exclusion criteria were, i) non-English papers, ii) questionable results. Results were categorized and selected appropriately. The reference list of each study was also screened. From a total summary of 103 manuscripts, 25 were finally included. All details regarding the selection of eligible studies are presented in Figure 1 (Prisma Flow diagram).

Anatomy results

**Figure 2:**
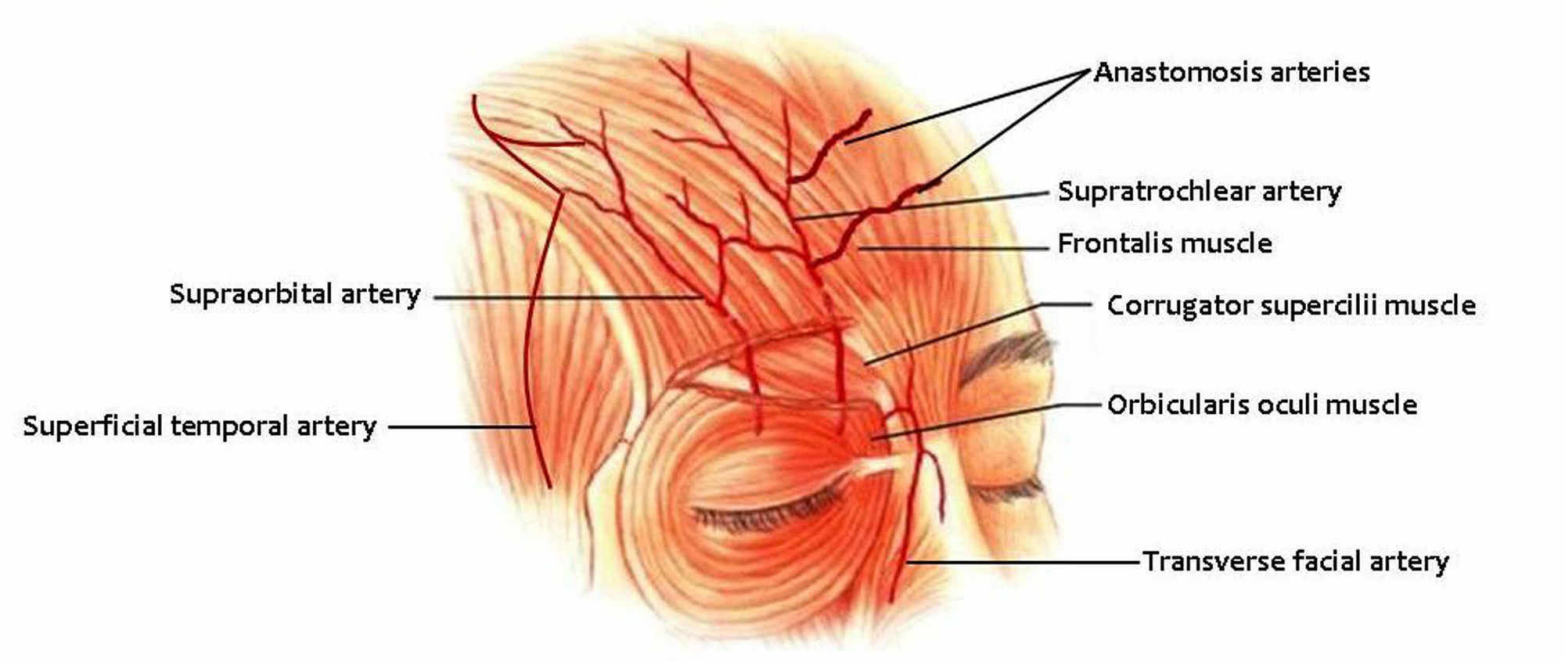
Supratrochlear artery and topographic anatomy A presentation of supratrochlear artery and topographic anatomy (Figure modified by authors).

In most of the cases, the STA usually emerges from the OA, a branch of the ICA [1]. It exits the orbit, as a separate vessel from the supraorbital artery (SOA) in 87% of the cases (Figure 2), or through the supratrochlear foramen (or notch), or over the supraorbital rim (SOR), by piercing the orbital septum approximately 1.7-2.2 cm from the midline and in a mean distance of 1.2 cm superior to the medial canthus (MC) [2,6-8]. Apart from the typical pattern, a small percentage of variations has been described regarding the origin of the STA. According to the available data, in about 12% of the cases, the STA and the SOA arise from the orbit as a single vessel. This vessel then bifurcates into two large branches which continue in a cephalad direction, following eventually the normal course of the SOA and the STA [6,7,9]. Kleintjes described two cases (2/60), where the STA was absent. In the first case, a branch from the angular artery (AA) named paracentral artery, provided a lateral branch to take over the arterial inflow associated with the STA at a paramedian position in the forehead. In the second case, a lateral branch from the paracentral artery was arisen. Then, after following a transverse course, it joined with the transverse frontal artery, a branch of the frontal branch of the superficial temporal artery [9]. Finally, Cong et al. reported a rare case where the STA arose directly from the AA [10].

A variety concerning STA's course is noted in various topographic descriptions. The STA, after exiting the orbit, passes superficial to the corrugator supercilii and deep to the orbicularis oculi and frontalis muscles [2,8]. Then, it ascends the forehead in a noticeably paramedian position, in a proximate distance of 2 cm from the midline [2]. More specifically, proximal to the corrugator supercilii muscle, the STA branches off to give rise to a superficial and a deep periosteal branch in a position 1.18 +/- 0.36 cm distal to the SOR and 1.35 +/- 0.34 cm lateral to the midline [11-13]. The superficial branch courses cephalad between the corrugator and the orbicularis oculi muscles and then pierces the frontalis muscle and becomes superficial to the later and the galea, traveling in the subcutaneous plane from 15-25 mm above the SOR or according to Potparic et al., at an average distance of 35 mm from the SOR [6,9,11,12,14]. Its average depth from the epidermal surface was found to be 1.5 mm [11]. On the other hand, the deep branch arises from the STA at about the level of the SOR. It then penetrates the corrugator supercilii muscle before it reaches the pericranium, where it travels in an axial fashion for 15-40 mm above the SOR within the deep layers of the subgaleal fascia [6,11]. However, there has been some reports where the deep branch of the STA was actually nonexistent [13, 15]. STA's average diameter was reported to be approximately 1 mm [9, 16,17]. Interestingly, Edizer et al. described a difference in the average diameter of STA between the right and left side of the same person with the first being at 0.8 mm whereas the second at 1.0 mm [8]. The average length of the STA from the point where it branches off from the OA to the point where it crosses the SOR was measured at 51.1 up to 51.2 mm [11].

Apart from STA's course variations, a plethora of branching patters exists in the literature. Kleintjes noted in his study of nine side branches of the STA, which are the medial communicating branch (MCB), the lateral communicating branch (LCB), the superior palpebral artery (SPA), the brow artery (BA), the periosteal branches (PB), the cutaneous branches (CB), the oblique branch (OB), the medial and lateral vertical branches (MLVB), and the single vertical branch (VB) [9]. Furthermore, the STA participates in a rich anastomotic network. It anastomoses with the AA, the terminal part of facial artery, in the nasoglabellar area where together with the SOA and their contralateral vessels form vascular arcades, allowing in this way the communication of the ICA with the external carotid artery (ECA) [7]. This network of anastomoses includes, apart from the STA, the SOA and the AA, also the infraorbital, the lateral nasal, the bilateral dorsal nasal and some small periosteal perforating arteries that supply the paranasal region [6, 11, 18]. Moreover, within the lateral forehead, the ST and SO vessels anastomose with the frontal branch of the superficial temporal artery (TA), an end branch of the ECA [6]. Last but not least, the paired STAs anastomose with each other via a number of horizontal unnamed arteries that cross the midline [2,13].

Risk results in filler injections’ application

The increasing use of filler injections may lead to a number of adverse events, with varying degrees of severity. Minor side effects, such as local skin inflammation, are common but not clinically important. Major ones, such as vision loss, skin necrosis, cutaneous granulomas or even cerebral infarction have also been reported [16-19]. The last one seems to be rather rare. The proposed mechanism for the aforementioned events is the direct arterial blood flow occlusion, resulting from the injected dermal filler which contains substances as hyaluronic acid, collagen or autologous fat. The occlusion of the artery leads to a reduction of blood flow to the area that the vessel normally supplies (skin, eye, brain) with corresponding results. This is actually considered to be the pathophysiological mechanism in the case of filler-induced ocular complications [19-22]. Perforator branches, such as the ones of the transverse facial artery, might help to avoid vessel injury and even occlusion.

The central retinal artery (CRA) is a branch of the OA and responsible for the blood supply of the retina along with the posterior ciliary arteries [23]. Direct cannulation of the STA in the nasoglabellar area, or at the medial part of the forehead, may lead to a retrograde course of the filler droplet to the OA, which could ultimately reach the CRA causing vascular occlusion [19-22]. This could be rather possible if the injection pressure exceeds the systolic arterial pressure [11, 24]. The average injection pressure needed for the OA to be embolized, has been measured to be 166.7 mmHg [11]. Meanwhile, the average volume of filler necessary to occupy the STA from the glabella to the bifurcation of the OA and the CRA is noted to be 0.085 ml [25]. Taking into consideration the unnamed arteries that anastomose with the pair of the STAs, we may assume that there is a possibility for the injection in one of them to cause a subsequent embolism, which may lead to bilateral ocular complications [26].

Anatomic findings have shown that the nasoglabellar region constitutes a volatile area to be injected as far as a needle-induced misadventure is concerned. The reason for that is the vascular relationship between the AA, the STA and the SOA which form a rich and inconsistent arterial plexus, and through their direct connection to the OA, a shorter pathway to the CRA [11,16]. Thus, a possible accidental vessel cannulation and embolization of the CRA could lead to a series of ocular complications [16].

Discussion

The STA represents a relatively small vessel with a short and a quite constant course. Although its anatomy is predictable, some anatomical branching variations have been described even between the two hemi-faces of the same person. Despite its relatively small size, the STA is important for the blood supply of the glabellar region and the medial aspect of the forehead. Additionally, via the rich anastomotic vascular plexus that is being formed between the STA, the SOA and the AA in the nasoglabellar area, the communication within the internal and the external carotid artery is feasible, magnifying the STA's role [1,7].

Recently a constantly increasing demand for minimally invasive cosmetic procedures and especially for soft tissue filler injections is being reported by various studies in the USA. Moreover, the most frequent area of application for filler injections is the facial. According to the American Society of Plastic Surgeons, soft tissue filler injections consist the second most popular procedure, whereas the first is botulinum toxin type A injection. Data imply that an increase of 3% has taken place from 2016 to 2017 and 2% from 2017 to 2018 with an overall huge increase of 312% from 2000 to 2017 [3]. Such an increase creates a parallel growth in risk number for adverse effects during aesthetic procedures [16-19]. Complications such as arterial embolism and occlusion are high probable even in cases of skilled injectors. Blindness and stroke may occur [27]. However, risk numbers remain limited. Beleznay et al. in their review registered only 98 cases of blindness secondary to facial filler injections. As stated in the same analysis, the highest risk injection sites responsible for ocular complications were, in decreasing order, the glabella (38.8%), the nasal region (25.5%), the nasolabial folds (13.3%) and the forehead (12.2%). The complications were more likely to occur with autologous fat injections (82.6%) compared to hyaluronic acid (HA) injections (8.7%).

The vasculature of the forehead area presents a rather conservative anatomical mapping. However, even a short vessel as the STA may cause serious complications if cannulated [28]. Although there is no algorithm that can be used, emergency treatment options in suspected intra-arterial injection of fillers may include topical nitropaste, low-dose aspirin, hyperbaric oxygen (if available), warm massage, prostaglandin E1 injection, and low-molecular-weight heparin, while anterior chamber paracentesis and intraarterial thrombolysis have not yet been studied for their outcomes [29]. It seems that for the moment, limiting the volume per injection could represent a simple prophylactic strategy [30].

Health care injectors should have a firm understanding of the vascular course of high-risk sites. Taking into consideration that the STA firstly arises deep in the superomedial orbit and then continues subcutaneously from 15 up to 25 mm above the SOR as it courses cephalad, injections at the glabella or inferior forehead at the level of the SOR or within 2 cm of that location should be performed as superficial as possible. On the other hand, injections in the upper forehead should be made under a periosteal plane in order to avoid intravascular cannulation, since the STA at this point takes its place at the subcutaneous plane [5]. Nevertheless, according to the study of Cho et al., the anterior branch of the STA has been indicated to be unexpectedly superficial lying 1.5 mm deep to the epidermal surface rendering the superficial injection a quite unsafe procedure [11].

## Conclusions

The STA is of vital importance and despite its quite small size, it plays a significant role in the injurious pathogenetic mechanism after soft-tissue injections. To minimize adverse effects during cosmetic injections in the frontal cranium area, the practitioner should have a deep knowledge of the local anatomy to mitigate danger of complications in the STA high-risk area and to deliver optimal aesthetic results with safety.
